# An Improved Quantum-Behaved Particle Swarm Optimization Algorithm with Elitist Breeding for Unconstrained Optimization

**DOI:** 10.1155/2015/326431

**Published:** 2015-05-10

**Authors:** Zhen-Lun Yang, Angus Wu, Hua-Qing Min

**Affiliations:** ^1^School of Computer Science and Engineering, South China University of Technology, Guangzhou 510006, China; ^2^School of Information Engineering, Guangzhou Panyu Polytechnic, Guangzhou 511483, China; ^3^Department of Electronic Engineering, City University of Hong Kong, Tat Chee Ave, Hong Kong

## Abstract

An improved quantum-behaved particle swarm optimization with elitist breeding (EB-QPSO) for unconstrained optimization is presented and empirically studied in this paper. In EB-QPSO, the novel elitist breeding strategy acts on the elitists of the swarm to escape from the likely local optima and guide the swarm to perform more efficient search. During the iterative optimization process of EB-QPSO, when criteria met, the personal best of each particle and the global best of the swarm are used to generate new diverse individuals through the transposon operators. The new generated individuals with better fitness are selected to be the new personal best particles and global best particle to guide the swarm for further solution exploration. A comprehensive simulation study is conducted on a set of twelve benchmark functions. Compared with five state-of-the-art quantum-behaved particle swarm optimization algorithms, the proposed EB-QPSO performs more competitively in all of the benchmark functions in terms of better global search capability and faster convergence rate.

## 1. Introduction

Particle swarm optimization (PSO), inspired by the social behavior of bird flocks [1], is an important and widely used population-based stochastic algorithm. Unlike evolutionary algorithms, PSO is computationally inexpensive and its implementation is straightforward. Each potential solution in PSO, represented by a particle, flies in a multidimensional search space with a velocity dynamically adjusted by the particle's own former information and the experience of the other particles. For its superiority, PSO has rapidly developed with applications in solving real-world optimization problems in recent years [2–5].

However, as demonstrated by van den Bergh [6], PSO is not a guaranteed global convergence algorithm according to the convergence criteria in [7]. Based on quantum mechanics and trajectory analysis of PSO [8], Sun et al. [9] proposed a variant of PSO, quantum-behaved PSO (QPSO) algorithm, which is theoretically proved to be global convergent using Markov process [10, 11]. The global convergence of QPSO guarantees to find the global optimal solution upon unlimited number of search iterations. Nevertheless, such condition is unrealistic when it comes to the real-world problems as only a finite number of iterations are allowed for the search of optimal solution on using any optimization algorithm. Thus, QPSO is also likely to be trapped in local optima or with slow convergence speed when it is used to solve complex problems. So far, many researchers developed various strategies to improve performance of QPSO in terms of convergence speed and global optimality [12–21]. However, it is rather difficult to improve the global search capability and accelerate the rate of convergence simultaneously. If any attempt focuses on avoiding being stuck at local optima, it is likely to have a slower convergence rate.

In PSO, the personal best (*pbest*) of each particle and global best (*gbest*) of the swarm found so far in the search process can be considered as the elitists of the whole swarm at any search iteration. In most of the current QPSOs, the information of elitists is either used directly or with some simple extra processing to guide the flying behavior of each particle in the search space; to the best of our knowledge, deep exploration with the elitist is not taken into account to assist the search of solutions in any work of QPSO reported in literatures. The exploration on the elitists will produce some extra information that may be beneficial for the search of the optimal solution.

In this study, a novel variant of the QPSO algorithm, called the quantum-behaved particle swarm optimization with elitist breeding (EB-QPSO), is proposed for the desirable aims to achieve better global search capability and convergence rate by employing a breeding scheme through transposon operators on elitists of the swarm. Elitist breeding is a kind of advanced elitist exploration method treating the elitist members as parents to create new diverse individuals with transposon operators. On one hand, elitist breeding helps to diversify the particle swarm during the search and thus enhance the global search capability of the algorithm. On the other hand, the new bred individuals with better fitness are selected as the new members of elitists and used to guide the swarm to perform exploration and exploitation more efficiently. Experiment results on twelve benchmark functions show that EB-QPSO outperforms the original QPSO and other four state-of-the-art QPSO variants.

The rest of this paper is organized as follows. A brief introduction of QPSO is presented in Section 2. In Section 3, an overview of related work is given. The proposed EB-QPSO algorithm is elaborated and compared with various existing QPSO algorithms over twelve benchmark functions in Sections 4 and 5, respectively. Finally, the general conclusions of the paper are given in Section 6.

## 2. Quantum-Behaved Particle Swarm Optimization

In the original PSO, each particle is defined by a position vector **x** = (*x*_1_, *x*_2_,…, *x*_*D*_) which signifies a solution in the search space and associated with a velocity vector **v** = (*v*_1_, *v*_2_,…, *v*_*D*_) responsible for the exploration of the search space. Let *N* denote the swarm size and *D* the dimensionality of the search space, during the evolutionary process, the velocity and the position of each particle are updated with the following rules:

(1) vi,dt+1=ωvi,dt+c1ri,dtpbesti,dt−xi,dt+c2Ri,dtgbestdt−xi,dt,xi,dt+1=xi,dt+vi,dt+1,

where *i*  (1 ≤ *i* ≤ *N*) and *d*  (1 ≤ *d* ≤ *D*), *v*_*i*,*d*_^*t*^ and *x*_*i*,*d*_^*t*^ are the *d*th dimension component of velocity and position of particle *i* in search iteration *t*, respectively, *pbest*_*i*,*d*_^*t*^ and *gbest*_*d*_^*t*^ are the *d*th dimension of the personal best of particle *i* and the global best of the swarm in search iteration *t*, respectively, *ω* is the inertia weight, *c*_1_ and *c*_2_ are two positive constant acceleration coefficients, and *r*_*i*,*d*_^*t*^ and *R*_*i*,*d*_^*t*^ are two random numbers uniformly distributed in the interval (0, 1).

According to the trajectory analysis given by Clerc and Kennedy [8], the convergence of the PSO algorithm may be achieved if each particle converges to its local attractor *p*_*i*_ = (*p*_*i*,1_, *p*_*i*,2_,…, *p*_*i*,*D*_), of which the coordinates are defined as

(2) $$\begin{array}{c} p_{i,d}^{t}=\varphi_{d}^{t}\times {pbest}_{i,d}^{t}+\left(\;1-\varphi_{d}^{t}\;\right)\times {gbest}_{d}^{t}, \end{array}$$

where *φ*_*d*_^*t*^ = *c*_1_ *r*_*i*,*d*_^*t*^/(*c*_1_ *r*_*i*,*d*_^*t*^ + *c*_2_ *R*_*i*,*d*_^*t*^).

The concept of the QPSO was developed based on the analysis above. Each single particle in QPSO is treated as a spin-less one moving in quantum space and the probability of the particle's appearing at position *x*_*i*_^*t*^ in the search iteration *t* is determined from a probability density function [22]. Employing the Monte Carlo method, each particle flies with the following rules:

(3) xi,dt+1pi,dt+αxi,dt−mbestdtln⁡⁡1ui,dt,if  rand⁡v≥0.5,xi,dt+1=pi,dt−αxi,dt−mbestdtln⁡⁡1ui,dt,if  rand⁡v<0.5,

where *α* is a parameter called contraction-expansion coefficient; both *u*_*i*,*d*_^*t*^ and rand⁡*v* are random numbers uniformly distributed on [0,1]; *mbest* is a global virtual point called mainstream or mean best defined as

(4) $$\begin{array}{c} {mbest}_{d}^{t}=\frac{1}{N}\overset{N}{\underset{i=1}{\sum }}{pbest}_{i,d}^{t}. \end{array}$$

A time-varying decreasing method [23] usually is adapted to control the contraction-expansion coefficient defined as follows:

(5) $$\begin{array}{c} \alpha =\alpha_{1}+\frac{\left(T-t\right)\times \left(\alpha_{0}-\alpha_{1}\right)}{T}, \end{array}$$

where *α*_0_ and *α*_1_ are the initial and final values of *α*, respectively; *T* is the maximum number of iterations; *t* is the current search iteration number.

The QPSO algorithm has simpler evolutional equation forms and fewer parameters than classical PSO, substantially facilitating the control and convergence in the search space. Without loss of generality, let *f* be the objective function to be minimized; the procedure for implementing the QPSO is given in Algorithm 1.

## 3. Related Work

Since QPSO was proposed, it has attracted much attention and different variants of QPSO have been proposed to enhance the performance from different aspects and applied to solve various real world optimization problems. In this section, a brief review of these QPSO variants will be presented. In general, most current QPSO variants can be classified into three categories, that is, the improvement based on operators from other evolutionary algorithms, hybrid search methods, and cooperative methods.

To improve the search efficiency, different operators derived from other evolutionary algorithms were introduced into QPSO algorithms. The mutation operator provides diversity in the search of solutions and consequently enhances the global search capability; therefore, various mutation operators based on Cauchy probability distribution [24], Gaussian probability distribution [14, 25], Lévy probability distribution [20], and chaotic sequences [26] were proposed to improve the QPSO performance in preventing premature convergence to local optima. Selection operators are beneficial in making good use of the information of elitists and the particles' position in the last iteration. Elitist selection operator and ranking strategy were introduced into QPSO to balance the convergence rate and global searching ability in [27, 28]. In [15], the* mbest* in each particle's position update procedure is determined by a random selection operator on all the* pbests* of the whole swarm. Another approach incorporating a ranking operator to select a random particle with its personal best position replacing the global best position in order to guide the particle to escape from the local optima is also proposed in [15]. Besides mutations and selections mentioned above, DE operators [29] and crossover operator [30] were also integrated into QPSO to enhance the particles' search capability.

Various search methods, including those from other optimization algorithms and the new proposed approaches, were incorporated into QPSO for better search efficiency. In [31], local search was incorporated into the main global search mechanism of QPSO to enhance the searching qualities of QPSO. The chaotic search method was integrated into QPSO to diversify the swarm in the latter period of the search process so as to alleviate the likelihood to be stuck in the local optima [32]. In [33, 34], the immune system was introduced to QPSO to effectively restrain the degeneration in the process of optimization. Simulated annealing was incorporated into QPSO to improve the ability to effectively jump out of the local optima in [35].

The third category, cooperative methods, refers to the searches performed with multiswarms or the approaches of optimizing different components of each solution vector separately. In [36], two subswarms were used to search in different layers and the cooperation of the subswarms leads to better performance in approximating solutions to the global optima. In [21, 37], a particle is split into several subparts and makes contribution to the population not only as a whole item but also in subset of the position vector.

## 4. The Proposed Algorithm

There are numerous variants of QPSO which have been proposed in recent literatures. In most of the QPSOs, elitists are simply stored in memory for the solution comparison or through simple processing step like mutation for the exploration search. Nevertheless, to the best of our knowledge, all the existing QPSOs seldom explore the elitist memory and get more potential information from it, which might be a significant trend to improve the performance of the algorithm.

The memory of QPSO mainly consists of the elitist individuals which are the personal best of each particle and the global best of the whole swarm. According to the iterative equation of QPSO, the memory is very important in affecting the behavior of the swarm and thus impinges the performance of the algorithm. If the personal best particles and the global best particle get trapped in local optima, the algorithm will likely converge to local optima. On the other hand, if the individuals in memory are less likely to be stuck at local optima, the algorithm can achieve better solutions.

In fact, we believe that the information from elitists can have a better use in aiding the search exploration and exploitation of the global optima. New subswarms can be generated from the elitists with proper mechanism to aim at the better search efficiency in terms of the solution quality and rate of convergence. An elitist exploration strategy, namely, elitist breeding, is proposed in this study. It makes use of the elitists generated in the evolutionary processes of algorithm to create new subswarms through the proposed breeding scheme. In the elitist breeding scheme, an elitist pool consists of personal best particles and global best particle found so far was constructed, and then the transposon operators which has the ability to enhance the diversity of solutions are selected as the breeding operators to explore the elitist memory and extract some more potential essences from the elitist individuals, thus to improve the search efficiency of QPSO. Moreover, the mechanism of updating the elitists with the new bred individuals with better fitness also provides a more efficient and precise search guidance for the swarm. The pseudocodes of the proposed EB-QPSO are given in Algorithm 2.

We can see from the pseudocodes of EB-QPSO that when the criteria is met, the personal best of each particle and the global best will be put into an elitist pool named* epool* and a new subswarm called* epool_eb* is generated through the transposon operators on the elitist pool. The size of* epool_eb* is the same as the* epool* and each individual in both of the pools is identified with its sequential order. To maintain the diversity of the swarm, each individual in* epool_eb* is only compared with the member with the same sequential order in* epool*. The* pbests* will be updated when the new generated individual is better than the corresponding one in* epool*. Here, a predefined parameter *λ* is used to control the frequency of elitist breeding. In every *λ* iteration, the breeding operation will be performed once.

**Figure 1:**
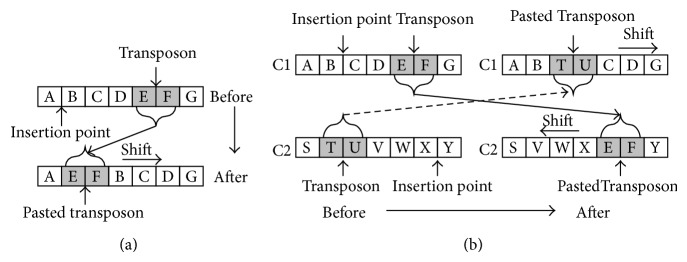
Cut-and-paste transposon operator. (a) Cut-and-paste in same chromosome. (b) Cut-and-paste in different chromosomes.

**Figure 2:**
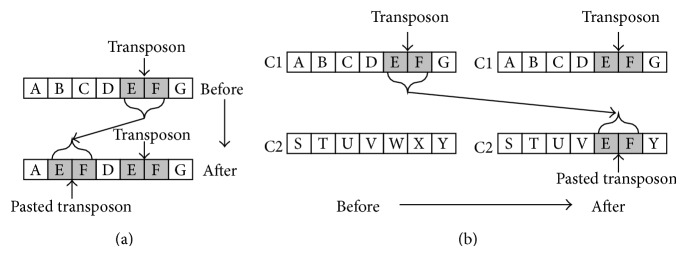
Copy-and-paste transposon operator. (a) Copy-and-paste in same chromosome. (b) Copy-and-paste in different chromosomes.

Transposon operators were firstly proposed by Man et al. [38] and mainly used in multiobjective evolutionary algorithms (MOEAs). A transposon is made of consecutive genes located in the randomly assigned position in each chromosome while the transposon operators are lateral movement operations that happen in one chromosome or between different ones. Generally speaking, there exist two types of transposon operators, that is, cut-and-paste and copy-and-paste, which are demonstrated in Figures 1 and 2. The transposon operations conducted within an individual chromosome or on a different chromosome are chosen randomly. Moreover, the size of each transposon can be greater than one and is decided by a parameter called jumping percentage while the number of transposons is also a predefined parameter. Another parameter, the jumping rate, is assigned to determine the probability of the activation of transposon operations.

**Figure 3:**
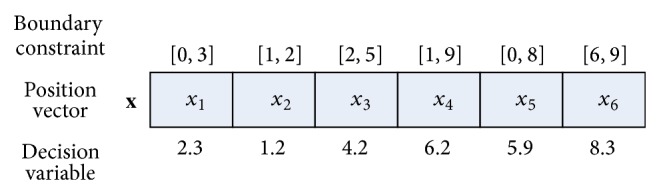
An example of the particle is represented as a chromosome.

As demonstrated in Figure 3, each particle which can be regarded as a chromosome consists of the same number of genes as the size of its position vector and each gene holds a real number of the corresponding decision variable. It is clear that the number of the chromosomes is the same as the number of particles in the swarm. With such representation, the transposon operators can be integrated into PSOs. However, since different decision variables might have different boundary constraints, the transposon operators might generate invalid individual. As illustrated in Figure 3, the boundary violation occurs if *x*_1_ is copied to the position of *x*_2_. To overcome the boundary violation problem caused by transposon operators, according to the description in [39], the position vector of each particle is normalized to a chromosome consists of the ratio value of each variable occupying in its own boundary range before performing the transposon operations. The conversion equation is defined as follows:

(6) $$\begin{array}{c} \text{conv}\left(\;x\;\right)=\frac{x-\text{lb}\left(x\right)}{\text{ub}\left(x\right)-\text{lb}\left(x\right)}, \end{array}$$

where lb(*x*) and ub(*x*) represent the lower and upper bounds of *x*, respectively. After the transposon operations, the value of each gene should be restored back to its corresponding positional value in the search space according to the equation as follows:

(7) $$\begin{array}{c} \text{resto}\left(\;x\;\right)=\text{conv}\left(\;x\;\right)\times \left(\;\text{ub}\left(\;x\;\right)-\text{lb}\left(\;x\;\right)\;\right)+\text{lb}\left(\;x\;\right). \end{array}$$

In transposon operations, a temporary pool called* epool_ratio* is used to store all the normalized chromosomes converted from the individuals in* epool* according to (6). When the transposon operations have been done, each individual will be restored from* epool_ratio* and saved to* epool_eb*. The pseudocodes of the function* transposon_op* are given in Algorithm 3.

## 5. Experiment Result and Discussion

To validate the performance of the proposed EB-QPSO, it was tested on a set of twelve widely adopted benchmark testing functions and compared with the original QPSO [9] and four state-of-the-art QPSO variants including WQPSO [28], CAQPSO [14], QPSO-RM [15] and QPSO-RO [15], which have been studied thoroughly in the corresponding literatures, on solution accuracy, convergence speed, and algorithm reliability. These QPSO variants are very competitive in comparing with some other forms of PSO algorithms including PSO with inertia weight (PSO-In) [40], PSO with constriction factor (PSOCo) [41], the Standard PSO [42], Gaussian PSO [43], Gaussian Bare Bones PSO [44], Exponential PSO (PSO-E) [45], Lévy PSO [46], comprehensive learning PSO (CLPSO) [47], dynamic multiple swarm PSO (DMS-PSO) [48], and fully informed particle swarm (FIPS) [49].

### 5.1. Test Instances and Algorithmic Configuration

The test set consisted of twelve unconstrained test instances widely used in early researches reported in literatures [50–53] as listed in Table 1 with seven unimodal functions (*f*_1_–*f*_7_) and five multimodal functions (*f*_8_–*f*_12_). These benchmark instances pose different difficulties to optimization algorithms. The more detailed description of each function can be found in [51]. Following [51], an “acceptance” value is defined to gauge the acceptability of a solution found by the QPSOs.

For the simulation experiment, the contraction expansion (CE) coefficients of the proposed EB-QPSO algorithm and the compared five QPSOs decreased linearly from *α*_0_ to *α*_1_ during the search process according to (5). *α*_0_ and *α*_1_ were set at 0.6 and 0.5 for EB-QPSO, QPSO-RM and QPSO-RO while 1.0 and 0.5 were used for QPSO, WQPSO, and CAQPSO accordingly. The other parameters of the proposed EB-QPSO algorithm for the experiment were chosen at values as given in Table 2. For the compared QPSO algorithms, parameters settings other than CE coefficients were used with values according to their corresponding references.

**Figure 4:**
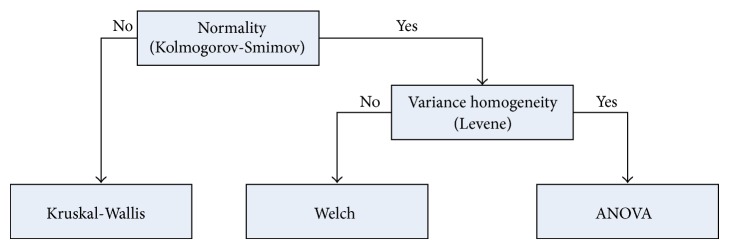
The general structure of the statistical analysis performed in this work.

For each test problem, 50 simulation trials were conducted for each of the compared algorithms with random initial populations. For a fair comparison among all the QPSO algorithms, they were tested with the same population size of 20 at each trial run. Furthermore, the maximum number of objective function evaluations (FEs) was set at 4.0 × 10^4^ for each test instance for all the algorithms. Since all the algorithms in the simulation experiment are stochastic, not solely compared on the success rate, it is essential to have the statistical analysis conducted so as to provide confidential comparisons. The median ($\tilde{x}$) and interquartile range (IQR) of each test instance were recorded as the measures of location (or central tendency) and statistical dispersion. To provide the confidential comparisons, the statistical analysis as in [54, 55] was used. The general structure of statistical analysis is given in Figure 4.

Kolmogorov-Smirnov test is firstly conducted to check whether the value of the results follow the normal (Gaussian) distribution or not. If the results do not follow the normal distribution, the nonparametric Kruskal-Wallis test is performed in order to compare the median result of each algorithm; if not, the Levene test is used to check the homogeneity of the variances. A Welch test is performed to verify the confidence of comparisons if the samples have different variance, otherwise ANOVA test is done to accomplish this task. The confidence level of 95% is used in the statistical analysis. The results are shown in the last column of Tables 3 and 4 with the symbol “+” indicating that the performance difference between the proposed EB-QPSO and best algorithm among the other QPSO algorithms is statistically significant, while the symbol “−” representing the difference is insignificant.

### 5.2. Comparisons on the Solution Accuracy

The performance results on the solution accuracy of each of the algorithms in the simulation experiment are shown in Table 3 in terms of the median and interquartile range of the solutions obtained in the 50 independent runs by each algorithm. The best result among those obtained by all six contenders for each problem is highlighted with boldface.

From the results in Table 3, we can see that the proposed EB-QPSO algorithm obtains the best values in all of the 12 test instances. It is worth mentioning that EB-QPSO achieves the solutions which have values reached or approximated to the global optima on problems *f*_3_, *f*_4_, *f*_7_, *f*_8_, and *f*_9_ whereas the other compared algorithms are unable to do so. It illustrates that the proposed EB-QPSO having the ability to avoid being stuck at the local optima with the benefits from the elitist breeding through transposon operators. The difference in performance of the proposed algorithm comparing to the other five QPSO is statistically significant as indicated in the statistical analysis result shown in the last column of the table.

### 5.3. Comparisons on the Convergence Speed and Reliability

**Figure 5:**
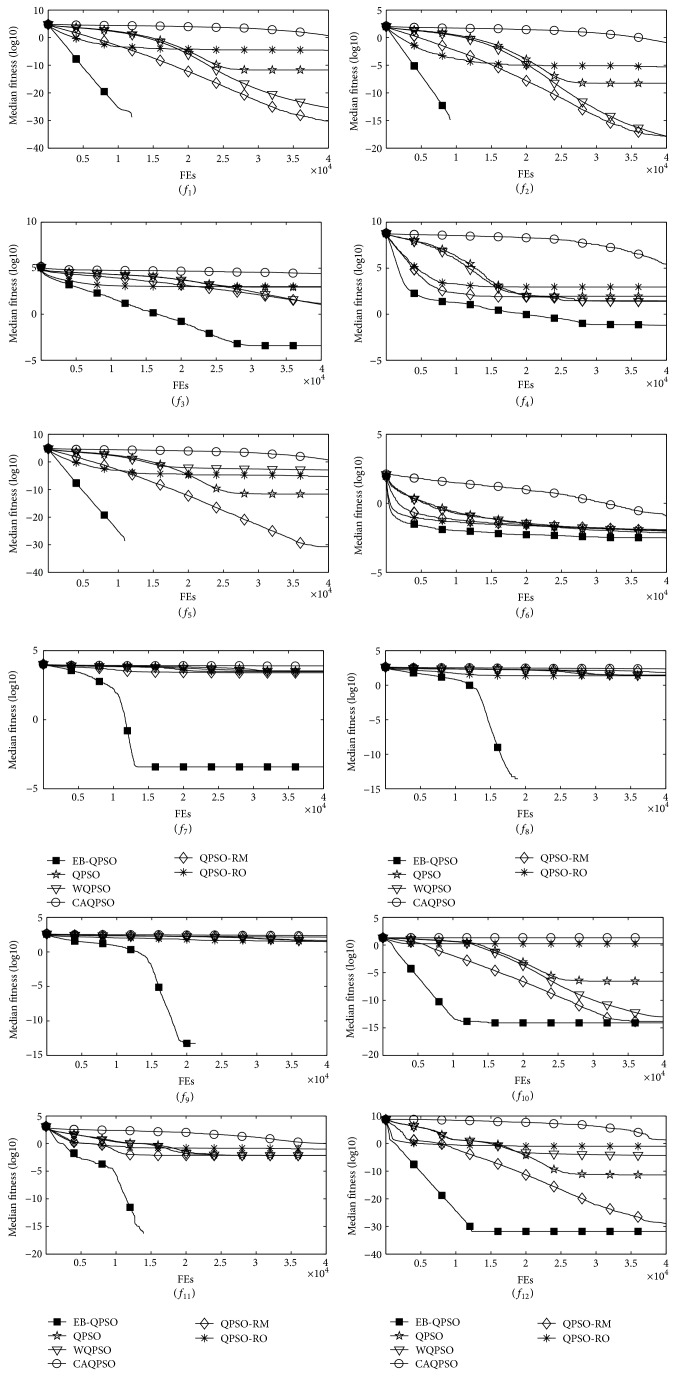
Convergence performance of the six QPSOs on the 12 test instances.

Another salient yardstick for evaluating the algorithm performance is the speed in approximating the global optimum. As shown in Table 4, EB-QPSO entirely offers a much higher convergence speed which is measured by the median and interquartile range of FEs number needed to reach an acceptable solution. Note that here “/” represents the corresponding algorithm cannot reach an acceptable solution in at least one-half of all the trials. To compare the convergence characteristics, Figure 5 graphically presents the convergence processes in terms of median results obtained in the 50 runs of all six contenders in solving the 12 test instances. It is worth mentioning that the global optima of all the test instances are at “0” and the logarithm of “0” has no mathematical meaning and cannot be displayed graphically. For any algorithm, if the optima is found, it will stop searching for solution further even though the maximum number of function evaluation limit is not reached; thus, upon such condition, the corresponding convergence curve for a particular test case will be stopped at that point, It is the case for problems *f*_1_, *f*_2_, *f*_5_, *f*_8_, *f*_9_, and *f*_11_ as the search convergences to the global optima. It shows that the proposed EB-QPSO has the best convergent efficiency amongst the compared algorithms in all 12 test instances.

Reliability here refers to the success rate; that is, the percentage of trial runs reaching acceptable solutions.  Table 5 reveals that EB-QPSO is able to reach acceptable solutions in all the trials over all the test instances whereas the compared algorithms cannot.

## 6. Conclusion

QPSO is a promising optimization technique which has shown its superiority in solving wide range of optimization problems. However, it is still a difficult problem to improve the global search capability and accelerate convergence speed of QPSO simultaneously. In this paper, we presented an improved quantum-behaved particle swarm optimization algorithm with elitist breeding (EB-QPSO) for unconstrained optimization. The novel elitist breeding scheme acts on the elitists found during the evolutionary process to jump out of the likely local optima and guide the swarm to perform exploration and exploitation more efficiently and thus improves the performance of QPSO in terms of better global search capability and faster convergence speed. The performance of EB-QPSO has been compared against the existing QPSO algorithms, the original, WQPSO, CAQPSO, QPSO-RM, and QPSO-RO on a test suite consisting of twelve benchmark functions. All simulation results have demonstrated that EB-QPSO has superiority over other QPSOs in solution accuracy, convergence speed, and reliability significantly. Besides, EB-QPSO can locate the global optima of most of the test functions while the other algorithms cannot. Our further work will concentrate on applying the EB-QPSO algorithm to the real-world optimization problems and on integrating the approach of elitist breed into other swarm intelligence algorithms. In this paper, the proposed EB-QPSO was studied empirically; the theoretical analysis of the global convergence of EB-QPSO will be developed in the future.

## Figures and Tables

**Algorithm 1:**
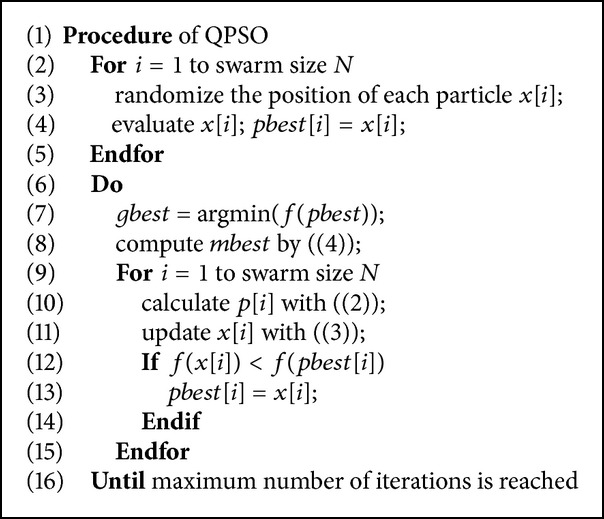
The pseudocodes for QPSO algorithm.

**Algorithm 2:**
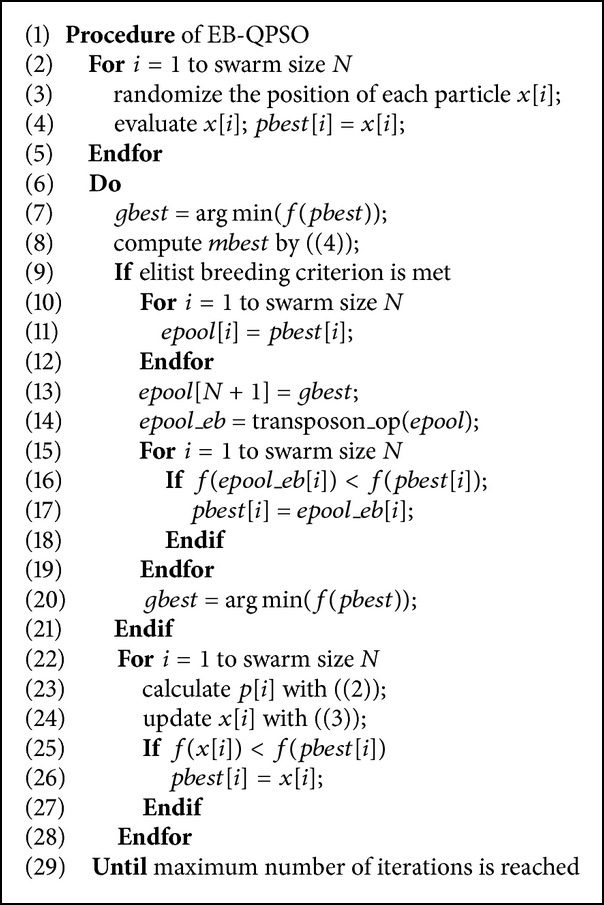
The pseudocodes for the proposed EB-QPSO algorithm.

**Algorithm 3:**
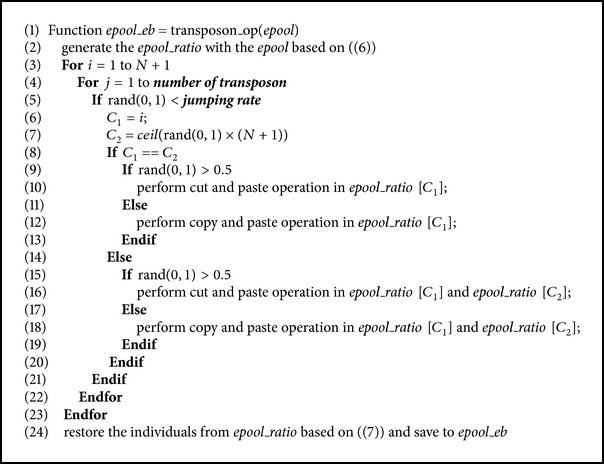
The pseudocodes for the transposon operations in the proposed algorithm.

**Table 1:** The test instances used in the experiment.

Name	Function	*D*	Search space	Global *f*_min⁡_	Acceptance
Sphere	*f*_1_(*x*) = ∑_*i*=1_^*D*^ *x*_*i*_^2^	30	[−100,100]^*D*^	0	0.01

Schwefel's P2.22	$f_{2}\left(x\right)=\sum_{i=1}^{D}\left|x_{i}\right|+\prod_{i=1}^{D}\left|x_{i}\right|$	30	[−10,10]^*D*^	0	0.01

Quadric	*f*_3_(*x*) = ∑_*i*=1_^*D*^(∑_*j*=1_^*i*^ *x*_*j*_)^2^	30	[−100,100]^*D*^	0	100

Rosenbrock	*f*_4_(*x*) = ∑_*i*=1_^*D*−1^[100(*x*_*i*+1_ − *x*_*i*_^2^)^2^ + (*x*_*i*_ − 1)^2^]	30	[−100,100]^*D*^	0	100

Step	*f*_5_(*x*) = ∑_*i*=1_^*D*^(|*x*_*i*_ + 0.5|)^2^	30	[−100,100]^*D*^	0	0

Quadric Noise	*f*_6_(*x*) = ∑_*i*=1_^*D*^ *ix*_*i*_^4^ + rand[0,1]	30	[−1.28,1.28]^*D*^	0	0.01

Schwefel	$f_{7}\left(x\right)=\sum_{i=1}^{D}-x_{i}\sin\left(\sqrt{x_{i}}\right)+12569.5$	30	[−500,500]^*D*^	0	2569.5

Rastrigin	*f*_8_(*x*) = ∑_*i*=1_^*D*^[*x*_*i*_^2^ − 10cos⁡(2π*x*_*i*_) + 10]	30	[−5.12,5.12]^*D*^	0	50

Noncontinuous Rastrigin	*f*_9_(*x*) = ∑_*i*=1_^*D*^[*y*_*i*_^2^ − 10cos⁡(2π*y*_*i*_) + 10]	30	[−5.12,5.12]^*D*^	0	50
	where $\;y_{i}=\left\{\begin{array}{cc} x_{i}, & \left|x_{i}\right|<0.5\\ \frac{\text{round}(2x_{i})}{2}, & \left|x_{i}\right|\geq 0.5 \end{array}\right.$				

Ackley	$f_{10}\left(x\right)\;=-20\exp\left(-0.2\sqrt{\frac{1}{D}\sum_{i=1}^{D}x_{i}^{2}}\right)$	30	[−32,32]^*D*^	0	0.01
	$-\exp\left(\frac{1}{D}\sum_{i=1}^{D}\cos2\pi x_{i}\right)\;+20+e$				

Griewank	$f_{11}\left(x\right)=\frac{1}{4000}\sum_{i=1}^{D}x_{i}^{2}-\prod_{i=1}^{D}\cos\left(\frac{x_{i}}{\sqrt{i}}\right)+1$	30	[−600,600]^*D*^	0	0.01

Generalized penalized	$f_{12}\left(x\right)\;=\frac{\pi }{D}\left\{10\;\sin^{2}\left(\pi y_{i}\right)+\sum_{i=1}^{D-1}{\left(y_{i}-1\right)}^{2}\left[1+10\sin\left(\pi y_{i+1}\right)\right]\right\}$	30	[−50,50]^*D*^	0	0.01
	*f*_12_(*x*) = + (*y*_*D*_ − 1)^2^ + ∑_*i*=1_^*D*^ *u*(*x*_*i*_, 10,100,4)				
	where $\;y_{i}=1+\frac{1}{4}(x_{i}+1),u(x,a,k,m)=\left\{\begin{array}{cc} k{\left(x_{i}-a\right)}^{m}, & x_{i}>a\\ 0, & -a\leq x_{i}\leq a\\ k{\left(-x_{i}-a\right)}^{m}, & x_{i}<a \end{array}\right.$				

**Table 2:** The parameters setting for the proposed EB-QPSO.

Parameter	Value
CE coefficient (*α*)	*α*_0_ = 0.6, α_1_ = 0.5
Jumping percentage	6
Jumping rate	0.1
Number of transposons	6

**Table 3:** Search result comparisons among six QPSOs.

Problems	EB-QPSO $\tilde{x}$ (IQR)	QPSO $\tilde{x}$ (IQR)	WQPSO $\tilde{x}$ (IQR)	CAQPSO $\tilde{x}$ (IQR)	QPSO-RM $\tilde{x}$ (IQR)	QPSO-RO $\tilde{x}$ (IQR)	
*f*_1_	0.000**E** + 00	1.974*E* − 12	4.373*E* − 26	5.271*E* + 00	6.074*E* − 31	2.649*E* − 05	+
	(0.000**E** + 00)	(5.003*E* − 12)	(1.039*E* − 25)	(6.794*E* + 00)	(2.290*E* − 29)	(4.408*E* − 04)	

*f*_2_	0.000**E** + 00	5.497*E* − 09	1.459*E* − 18	1.225*E* − 01	1.319*E* − 18	5.124*E* − 06	+
	(0.000**E** + 00)	(1.344*E* − 08)	(2.283*E* − 18)	(5.969*E* − 02)	(7.520*E* − 18)	(8.130*E* − 05)	

*f*_3_	3.919**E** − 04	8.796*E* + 02	1.140*E* + 01	2.620*E* + 04	1.493*E* + 01	9.875*E* + 02	+
	(2.019**E** − 03)	(3.557*E* + 02)	(1.122*E* + 01)	(6.306*E* + 03)	(1.476*E* + 01)	(7.522*E* + 02)	

*f*_4_	6.290**E** − 02	8.830*E* + 01	2.476*E* + 01	2.643*E* + 05	2.879*E* + 01	8.907*E* + 02	+
	(1.828**E** − 01)	(1.324*E* + 02)	(6.817*E* + 01)	(5.027*E* + 05)	(1.004*E* + 02)	(1.238*E* + 03)	

*f*_5_	0.000**E** + 00	2.178*E* − 12	1.209*E* − 03	5.168*E* + 00	1.972*E* − 31	5.042*E* − 06	+
	(0.000**E** + 00)	(1.149*E* − 11)	(2.756*E* − 04)	(6.310*E* + 00)	(5.276*E* − 30)	(3.926*E* − 05)	

*f*_6_	3.138**E** − 03	7.314*E* − 03	9.824*E* − 03	1.219*E* − 01	1.096*E* − 02	1.246*E* − 02	+
	(2.605**E** − 03)	(3.798*E* − 03)	(6.296*E* − 03)	(7.925*E* − 02)	(5.525*E* − 03)	(9.904*E* − 03)	

*f*_7_	3.818**E** − 04	3.432*E* + 03	3.273*E* + 03	7.901*E* + 03	2.496*E* + 03	3.020*E* + 03	+
	(1.819**E** − 12)	(8.739*E* + 02)	(8.595*E* + 02)	(4.248*E* + 02)	(5.330*E* + 02)	(5.892*E* + 02)	

*f*_8_	0.000**E** + 00	2.933*E* + 01	2.600*E* + 01	2.335*E* + 02	6.847*E* + 01	2.289*E* + 01	+
	(0.000**E** + 00)	(1.344*E* + 01)	(6.959*E* + 00)	(3.252*E* + 01)	(7.292*E* + 01)	(7.699*E* + 00)	

*f*_9_	0.000**E** + 00	4.525*E* + 01	3.018*E* + 01	2.288*E* + 02	1.410*E* + 02	3.250*E* + 01	+
	(0.000**E** + 00)	(1.901*E* + 01)	(1.617*E* + 01)	(3.662*E* + 01)	(4.711*E* + 01)	(1.113*E* + 01)	

*f*_10_	7.994**E** − 15	2.774*E* − 07	1.004*E* − 13	2.023*E* + 01	1.510*E* − 14	1.712*E* + 00	+
	(7.105**E** − 15)	(5.001*E* − 07)	(4.263*E* − 14)	(1.035*E* + 01)	(1.243*E* − 14)	(1.252*E* + 00)	

*f*_11_	0.000**E** + 00	7.396*E* − 03	7.475*E* − 03	1.045*E* + 00	7.396*E* − 03	1.015*E* − 01	+
	(0.000**E** + 00)	(1.907*E* − 02)	(1.671*E* − 02)	(5.193*E* − 02)	(1.232*E* − 02)	(1.938*E* − 01)	

*f*_12_	1.571**E** − 32	4.510*E* − 12	4.557*E* − 05	2.077*E* + 01	1.057*E* − 29	1.039*E* − 01	+
	(0.000**E** + 00)	(4.574*E* − 11)	(1.012*E* − 05)	(6.941*E* + 01)	(9.400*E* − 27)	(4.960*E* − 01)	

**Table 4:** The FEs number needed to reach an acceptable solution for six QPSOs.

Problems	EB-QPSO $\tilde{x}$ (IQR)	QPSO $\tilde{x}$ (IQR)	WQPSO $\tilde{x}$ (IQR)	CAQPSO $\tilde{x}$ (IQR)	QPSO-RM $\tilde{x}$ (IQR)	QPSO-RO $\tilde{x}$ (IQR)	
*f*_1_	2.220**E** + 03	1.720*E* + 04	1.647*E* + 04	/	8.910*E* + 03	6.580*E* + 03	+
	(1.800**E** + 02)	(6.100*E* + 02)	(5.000*E* + 02)	(0.000*E* + 00)	(6.400*E* + 02)	(2.860*E* + 03)	

*f*_2_	2.230**E** + 03	1.694*E* + 04	1.593*E* + 04	/	9.140*E* + 03	4.700*E* + 03	+
	(1.400**E** + 02)	(6.950*E* + 02)	(3.600*E* + 02)	(0.000*E* + 00)	(6.750*E* + 02)	(9.450*E* + 02)	

*f*_3_	9.230**E** + 03	/	3.323*E* + 04	/	3.206*E* + 04	/	+
	(2.815**E** + 03)	(0.000*E* + 00)	(2.275*E* + 03)	(0.000*E* + 00)	(3.995*E* + 03)	(0.000*E* + 00)	

*f*_4_	4.730**E** + 03	1.743*E* + 04	1.788*E* + 04	/	9.190*E* + 03	/	+
	(1.470**E** + 03)	(1.957*E* + 04)	(3.900*E* + 03)	(0.000*E* + 00)	(1.214*E* + 04)	(0.000*E* + 00)	

*f*_5_	1.095**E** + 04	/	/	/	/	/	+
	(1.075**E** + 03)	(0.000*E* + 00)	(0.000*E* + 00)	(0.000*E* + 00)	(3.667*E* + 04)	(0.000*E* + 00)	

*f*_6_	1.099**E** + 04	2.974*E* + 04	2.563*E* + 04	/	/	/	+
	(7.035**E** + 03)	(8.495*E* + 03)	(3.415*E* + 04)	(0.000*E* + 00)	(3.366*E* + 04)	(0.000*E* + 00)	

*f*_7_	5.200**E** + 03	/	/	/	5.510*E* + 03	/	+
	(9.500**E** + 02)	(0.000*E* + 00)	(0.000*E* + 00)	(0.000*E* + 00)	(1.420*E* + 04)	(0.000*E* + 00)	

*f*_8_	4.700**E** + 03	2.920*E* + 04	2.749*E* + 04	/	/	1.008*E* + 04	+
	(8.000**E** + 02)	(3.910*E* + 03)	(2.985*E* + 03)	(0.000*E* + 00)	(3.404*E* + 04)	(6.340*E* + 03)	

*f*_9_	3.400**E** + 03	3.073*E* + 04	3.384*E* + 04	/	/	2.092*E* + 04	+
	(6.000**E** + 02)	(3.500*E* + 04)	(6.245*E* + 03)	(0.000*E* + 00)	(0.000*E* + 00)	(1.468*E* + 04)	

*f*_10_	2.490**E** + 03	1.809*E* + 04	1.726*E* + 04	/	1.021*E* + 04	/	+
	(2.050**E** + 02)	(6.800*E* + 02)	(7.250*E* + 02)	(0.000*E* + 00)	(9.850*E* + 02)	(0.000*E* + 00)	

*f*_11_	4.220**E** + 03	1.865*E* + 04	1.742*E* + 04	/	1.043*E* + 04	/	+
	(2.130**E** + 03)	(1.997*E* + 04)	(1.969*E* + 04)	(0.000*E* + 00)	(1.160*E* + 04)	(0.000*E* + 00)	

*f*_12_	2.010**E** + 03	1.754*E* + 04	1.735*E* + 04	/	9.500*E* + 03	/	+
	(4.350**E** + 02)	(1.400*E* + 03)	(1.785*E* + 03)	(0.000*E* + 00)	(2.230*E* + 03)	(8.655*E* + 03)	

**Table 5:** Reliability comparisons among six QPSOs.

Problems	EB-QPSO	QPSO	WQPSO	CAQPSO	QPSO-RM	QPSO-RO
*f*_1_	100.0%	100.0%	100.0%	0.0%	100.0%	100.0%
*f*_2_	100.0%	100.0%	100.0%	0.0%	100.0%	98.0%
*f*_3_	100.0%	0.0%	100.0%	0.0%	100.0%	0.0%
*f*_4_	100.0%	56.0%	76.0%	0.0%	68.0%	2.0%
*f*_5_	100.0%	0.0%	0.0%	0.0%	28.0%	0.0%
*f*_6_	100.0%	82.0%	52.0%	0.0%	42.0%	22.0%
*f*_7_	100.0%	4.0%	10.0%	0.0%	52.0%	18.0%
*f*_8_	100.0%	98.0%	98.0%	0.0%	40.0%	100.0%
*f*_9_	100.0%	62.0%	88.0%	0.0%	0.0%	92.0%
*f*_10_	100.0%	100.0%	100.0%	0.0%	100.0%	12.0%
*f*_11_	100.0%	62.0%	58.0%	0.0%	68.0%	4.0%
*f*_12_	100.0%	90.0%	96.0%	0.0%	88.0%	44.0%
